# Ease of sutureless aortic valve replacement in a patient with unexpected ochronosis: a case report

**DOI:** 10.1186/s13019-024-02834-4

**Published:** 2024-06-25

**Authors:** Saeid Hosseini, Soheila Salari, Mohammad Javad Alemzadeh Ansari, Mahshid Hesami, Sepideh Banar, Mohammad Amin Shojaei

**Affiliations:** 1 Heart Valve Diseases Research Center, Rajaie Cardiovascular Medical and Research Center, Iran University of Medical Sciences, Tehran, Iran; 2 Cardiovascular Intervention Research Center, Rajaie Cardiovascular Medical and Research Center, Iran University of Medical Sciences, Tehran, Iran; 3 Pathology Department, Rajaie Cardiovascular Medical and Research Center, Iran University of Medical Sciences, Tehran, Iran; 4 Heart Valve Diseases Research Center, Rajaie Cardiovascular Medical and Research Center, School of Medicine, Iran University of Medical Sciences, Tehran, Iran

**Keywords:** Alkaptonuria, Cardiac ochronosis, Aortic stenosis, Perceval^™^, Sutureless aortic valve replacement

## Abstract

**Background:**

Alkaptonuria is a rare congenital metabolic disorder characterized by homogentisic acid accumulation in body cartilage and connective tissues due to a deficient homogentisic acid dioxygenase enzyme. This disorder manifests in various clinical symptoms, including spondyloarthropathy, ocular and dermal pigmentation, genitourinary tract obstruction by ochronosis stones, and cardiovascular system involvement. Cardiac ochronosis is a rare manifestation of alkaptonuria that may present as aortic stenosis, sometimes accompanied by other cardiovascular complications.

**Case presentation:**

We report an unexpected case of ochronosis diagnosed during cardiac surgery. Due to the fragile, thin, and atheromatous nature of the ascending aorta in patients with ochronosis, we opted for a sutureless aortic valve replacement procedure. This approach appears to be more suitable for patients with ochronosis.

**Conclusions:**

Although cardiac ochronosis is rare, surgeons should remain vigilant and consider the possibility of this condition when examining patients with aortic valve stenosis, paying close attention to the clinical manifestations of alkaptonuria.

**Supplementary Information:**

The online version contains supplementary material available at 10.1186/s13019-024-02834-4.

## Introduction

Alkaptonuria is an autosomal recessive inherited disorder of tyrosine metabolism caused by a deficiency in homogentisic acid (HGA) dioxygenase, the third enzyme in the tyrosine degradation pathway. This deficiency results in HGA accumulation [1, 2]. In patients with alkaptonuria, HGA remains in circulation, with its oxidation products polymerized and deposited in cartilage and connective tissues. The three salient features of this disease are the darkening of urine upon exposure to air, ochronosis (dark pigmentation of the connective tissue), and degenerative arthritis [2, 3].

The deposition of bluish pigments can lead to aortic stenosis, the primary cardiovascular manifestation of ochronosis. Bluish pigmentation involves the heart valves, endocardium, intima of the aorta, pericardium, and coronary arteries. Pigmentation may also be observed in atherosclerotic plaques and areas of myocardial fibrosis [4, 5]. Aortic stenosis is the most frequently reported cardiac abnormality, with case series describing an increased prevalence compared with the general population [6].

In this report, we describe a case of ochronosis incidentally diagnosed during heart surgery. Considering the fragile atheromatous plaques in the thin-walled aorta, we selected a sutureless bioprosthetic valve for aortic valve implantation.

## Case presentation

A 55-year-old woman with a history of hypertension and diabetes mellitus was diagnosed with severe aortic valvular stenosis during preoperative investigations for total hip replacement. She was referred to our center for aortic valve replacement (AVR). Her past medical history included lumbar and cervical polyarthritis and a cholecystectomy. The patient’s weight was 47 kg, body mass index was 23 kg/m^2^, and body surface area was 1.3.

Transthoracic echocardiography confirmed a calcified aortic valve with severe stenosis (the mean pressure gradient: 40 mmHg, the peak pressure gradient: 60 mmHg, the aortic valve area (AVA): 0.87 cm^2^, and the AVA index: 0.66), mild aortic regurgitation, normal ejection fraction (55%), and mild concentric left ventricular hypertrophy. A small patent foramen ovale (size: 0.5 mm) was also observed. Cardiac catheterization revealed double-vessel coronary artery disease, affecting the mid-portion of the left anterior descending and circumflex coronary arteries. The patient was scheduled for open-heart surgery.

**Fig. 1:**
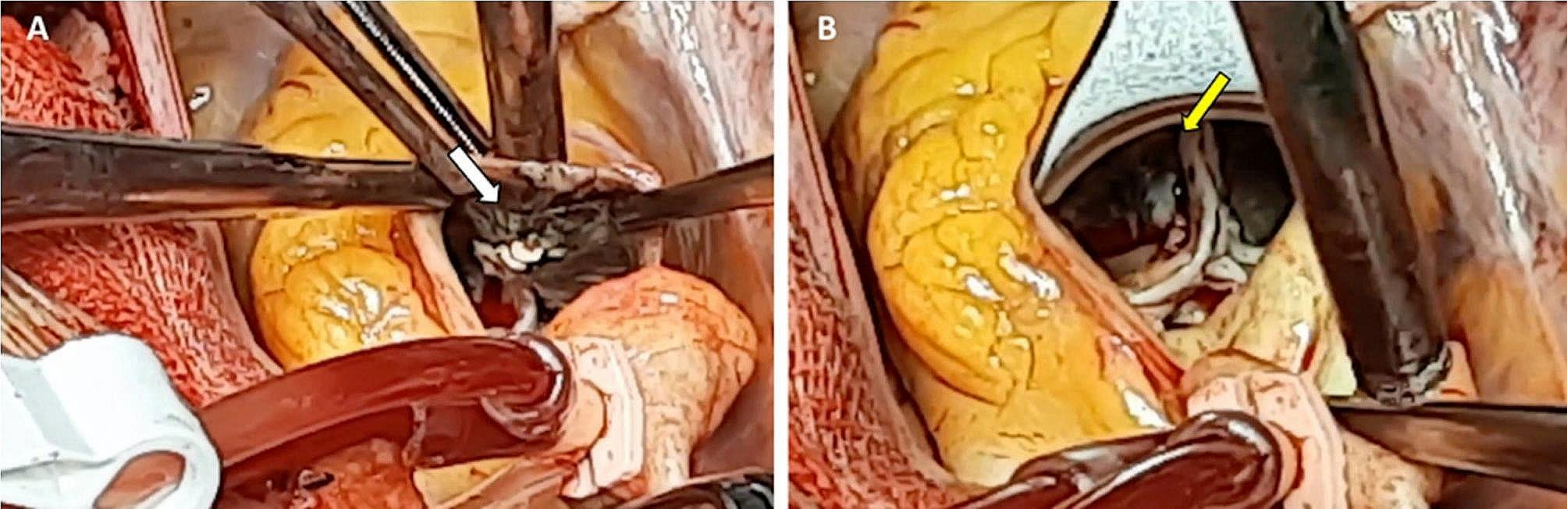
**(A**) The image shows a thin aortic wall with fragile, calcified, and dark plaques (the white arrow). (**B**) The image depicts a discolored ascending aorta (the yellow arrow)

Upon sternotomy, black pigmentation of the ascending aorta was observed. Following aortotomy, the tricuspid aortic valve was exposed and excised; it was thick, calcified, and displayed darkish pigmentation. Through the left ventricular outflow tract, the anterior mitral valve leaflets presented with similar pigmentation. The aortic wall was thin with fragile, blackish, calcified plaques (Fig. 1 & Video 1).

The patient underwent coronary artery bypass grafting with the left internal mammary artery on the left anterior descending and a saphenous vein graft on the obtuse marginal artery. Subsequently, a medium-sized Perceval (Corcym Co, London, UK) sutureless AVR procedure was performed, and the patent foramen ovale was closed. The patient was weaned off cardiopulmonary bypass uneventfully and transferred to the ICU. Postoperative echocardiography confirmed that the bioprosthetic aortic valve (Perceval) was in a good position, exhibiting good hemodynamic function (the pressure gradient: 20 mmHg, the mean gradient: 8.5 mmHg, and Aortic Accelartion Time( 68 ms) without paravalvular leakage.

**Fig. 2:**
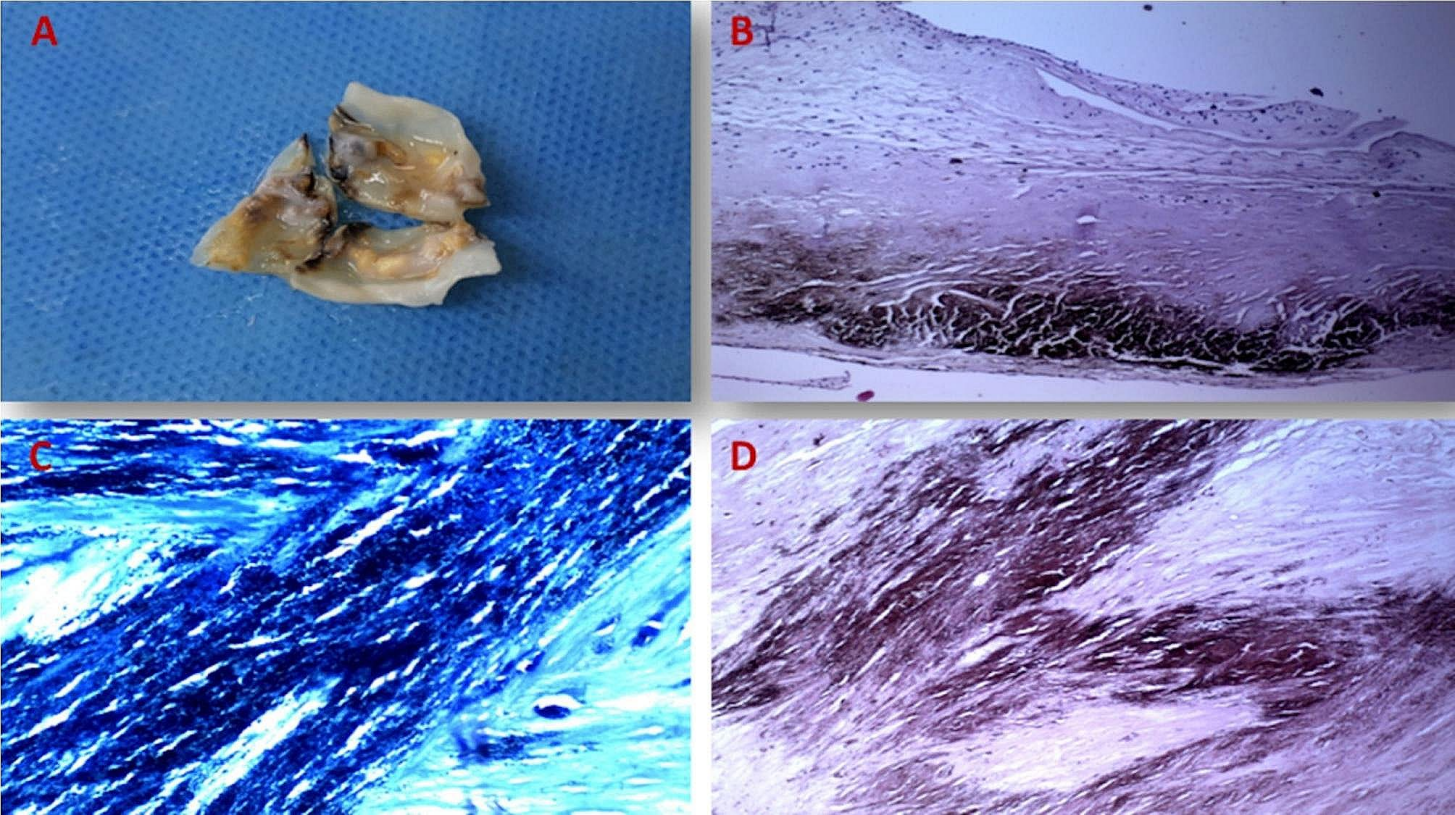
(**A**) Resected aortic cusps exhibit calcification and focal areas of dark discoloration. (**B**) Hematoxylin and Eosin (H&E) stained sections show valve tissue with calcification, fibrohyalinization, and foci of brown pigment deposition. (**C**) The deposits appear blue-black when stained with methylene blue. (**D**) Pigmentation is negative for iron, as shown by a Special Perls stain

In the ICU, the patient’s dark urine was observed, leading to the hypothesis of ochronosis. Upon further examination, blackish pigmentation was found in the temporal half of the sclerae and ear cartilages. Consequently, endogenous alkaptonuria and cardiac ochronosis were strongly suspected. Histological analysis of the aortic leaflet tissue confirmed the diagnosis (Fig. 2).

## Discussion

Alkaptonuria is an exceedingly rare congenital metabolic disorder, affecting 1 in 250,000 to 1 million births. The highest incidence is reported in Slovakia and the Dominican Republic, with a rate of 1 in 19,000 births [7].

In 1908, Sir Archibald Garrod recognized alkaptonuria as the first human disorder conforming to Mendelian principles of autosomal recessive inheritance [8].

Ochronosis, one of the manifestations of alkaptonuria, was first described by Virchow in 1866, drawing immediate attention among physicians due to the characteristic staining of various body tissues [9].

The disease usually manifests later in life, after the fourth decade, as renal clearance decreases with age. Clinical presentations exhibit wide variability. The most common complication is severe ochronotic spondyloarthropathy, impairing posture and gait. Nearly all patients require orthopedic surgery, with a mean age of 55 years for joint replacement, as observed in our patient, who had lumbar and cervical polyarthritis and was a candidate for total hip replacement [10, 11].

Other clinical manifestations include ocular and dermal pigmentation, as seen in our patient. Patients may also experience urogenital obstruction by ochronosis stones and cardiovascular system involvement, where polymerized HGA deposits in the aortic intima, aortic and mitral valves, coronary arteries, sub endocardium, and pericardium [6, 10].

Ochronotic deposition is primarily found in regions of turbulent flow, such as the sinotubular junction, which contributes to diastolic coronary filling. This explains the deposition of ochronotic pigment in the ostia of coronary arteries and aortic valve leaflets, while minimal deposition occurs in the venous circulation. The dynamics of vascular flow determine the site of pigment deposition, leading to subsequent microvascular damage [12].

.

Gaines and Pai [13] suggested that ochronotic pigment contributed to the formation of dystrophic aortic valve calcifications and stenosis. Aortic valve stenosis is the most frequent cardiac complication in alkaptonuria, with an increased prevalence in the sixth decade [6]. Other heart valves are typically unaffected, and dysfunction is rarely reported [10, 11].

In a study of patients with alkaptonuria, Hwida et al. [14] found a moderate correlation between the severity of aortic valve disease and joint involvement. However, the authors observed no correlation between urinary HGA levels and major cardiovascular risk factors.

Coronary artery involvement is common among patients with alkaptonuria, as seen in our patient, who exhibited double-vessel disease. In a recent study, patients with alkaptonuria did not show coronary artery calcification before age 40. Still, 50% of them presented with computed tomographic evidence of coronary artery calcification by age 59. Additionally, no correlation was found between coronary artery calcification and elevated serum cholesterol levels [7]. Given the age of onset for valvular and coronary complications in this disease, echocardiographic evaluation in the fourth decade and coronary computed tomography angiography in the fifth decade may aid in early diagnosis and successful treatment.

Currently, there is no approved treatment for alkaptonuria. Attempts at treatment with high-dose vitamin C and dietary restriction of tyrosine and phenylamine failed to reduce HGA levels and were unsuccessful [15]. The only effective treatment for reducing HGA levels in alkaptonuria is nitisinone, a potent inhibitor of the second enzyme in tyrosine catabolism [16].

No specific treatment guidelines are available for cardiovascular involvement in alkaptonuria. The choice of valve prosthesis during surgery remains uncertain; nonetheless, there are no reports of premature deterioration of bioprosthetic valves in these patients [17, 18]. For our patient, we opted for AVR with a sutureless Perceval aortic bioprosthetic valve to minimize interference with fragile atherosclerotic plaques in the ascending aorta, reducing the risk of embolization. This method also allows for faster implantation without the need for suturing [18].

The decision between a mechanical and bioprosthetic valve is typically based on the patient’s preference. Generally, for patients in this age range, below 60 years old, a mechanical prosthetic valve is recommended. Nevertheless, in this case, we performed a sutureless AVR procedure to minimize the risk of aortic wall damage and prevent embolization of atheromatous plaques.

## Conclusions

Despite its rarity, surgeons should consider the possibility of cardiac ochronosis when examining a patient with aortic valve stenosis and be attentive to the clinical manifestations of alkaptonuria. Considering the significance of selecting the appropriate valve type in patients with a fragile aortic wall and calcified atheroma, the Perceval valve may be a suitable, low-risk option (due to the risk of embolization of calcifications in the ring and wall during valve replacement) for AVR, specifically when the patient is not an ideal candidate for transcatheter AVR.

Furthermore, valve-in-valve procedures have been reported to be safely performed with the Perceval valve. Thus, in cases of valve degeneration, there is a viable option for future patient management.

### Electronic supplementary material

Below is the link to the electronic supplementary material.

Supplementary Material 1

## Data Availability

The data sets used and/or analyzed during the current study are available from the corresponding author upon reasonable request.

## Abbreviations

AVR: aortic valve replacement
HGA: homogentisic acid
AVR: aortic valve replacement
