# Supplementary material for: Ease of sutureless aortic valve replacement in a patient with unexpected ochronosis: a case report
Source: J Cardiothorac Surg. 2024 Jun 25;19:374. doi: 10.1186/s13019-024-02834-4 (PMC11197252; doi:10.1186/s13019-024-02834-4)
Supplement: Supplementary file 1 — Supplementary Material 1 [file 13019_2024_2834_MOESM1_ESM.docx]

**Video legend**


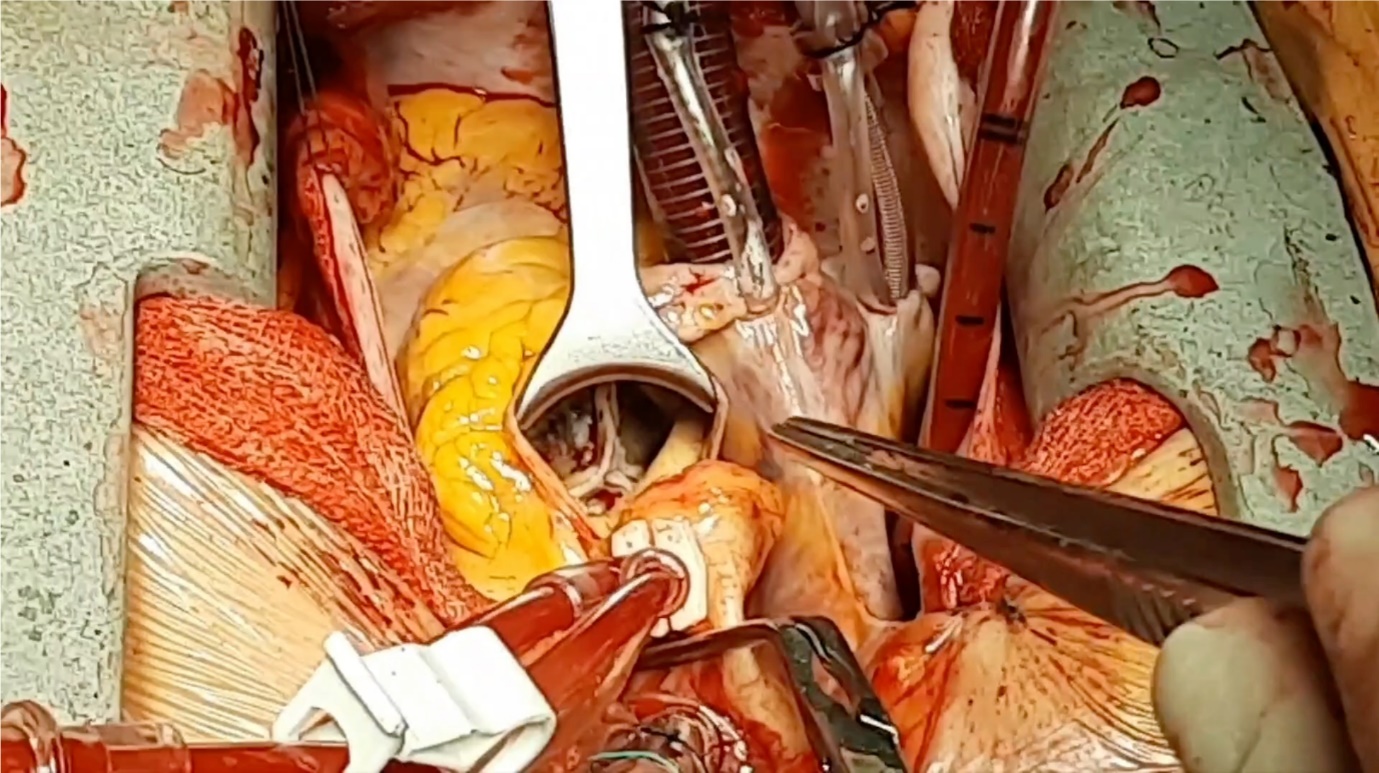


**Video 1:** The video shows a discolored ascending aorta featuring thin, fragile, calcified, and dark plaques.

**https://uupload.ir/view/untitled_(1)_1_1_1_3rzn.mp4/**
